# In vitro anti-*Leishmania* activity of tetracyclic iridoids from *Morinda lucida*, benth

**DOI:** 10.1186/s41182-016-0026-5

**Published:** 2016-08-05

**Authors:** Michael Amoa-Bosompem, Mitsuko Ohashi, Mba-Tihssommah Mosore, Jeffrey Agyapong, Nguyen Huu Tung, Kofi D. Kwofie, Frederick Ayertey, Kofi Baffuor-Awuah Owusu, Isaac Tuffour, Philip Atchoglo, Georgina I. Djameh, Faustus A. Azerigyik, Senyo K. Botchie, William K. Anyan, Regina Appiah-Opong, Takuhiro Uto, Osamu Morinaga, Alfred. A. Appiah, Irene Ayi, Yukihiro Shoyama, Daniel A Boakye, Nobuo Ohta

**Affiliations:** 1 Noguchi Memorial Institute for Medical Research, College of Health Sciences, University of Ghana, P. O. Box LG 581, Legon, Ghana; 2 Section of Environmental Parasitology, Faculty of Medicine, Tokyo Medical and Dental University, 1-5-45 Yushima, Bunkyo-ku, Tokyo, 113-8510 Japan; 3 Faculty of Pharmaceutical Sciences, Nagasaki International University, 2825-7 Huis Ten Bosch, Sasebo, Nagasaki 859-3298 Japan; 4 Centre for Plant Medicine Research, P. O. Box 73, Mampong - Akuapem, Ghana

**Keywords:** *Leishmania*, Leishmaniasis, Cytokinesis, Morphology, Proliferation, Microscopy, Chemotherapy

## Abstract

Leishmaniasis is an infectious disease transmitted by the sand fly. It is caused by over 20 different species of *Leishmania* and has affected over 14 million people worldwide. One of the main forms of control of leishmaniasis is chemotherapy, but this is limited by the high cost and/or toxicity of available drugs. We previously found three novel compounds with an iridoid tetracyclic skeleton to have activity against trypanosome parasites. In this study, we determined the activity of the three anti-trypanosome compounds against *Leishmania* using field strain, 010, and the lab strain *Leishmania hertigi*. The minimum inhibitory concentration (MIC) of the compounds against 010 was determined by microscopy while the IC_50_ of compounds against *L. hertigi* was determined by fluorescence-activated cell sorting with Guava viacount analysis. We found two of the three compounds, *molucidin* and *ML-F52*, to have anti-*Leishmania* activity against both strains. The fluor-microscope observation with DAPI stain revealed that both Molucidin and ML-F52 induced abnormal parasites with two sets of nucleus and kinetoplast in a cell, suggesting that compounds might inhibit cytokinesis in *Leishmania* parasites. Molucidin and ML-F52 might be good lead compounds for the development of new anti-*Leishmania* chemotherapy.

## Introduction

*Leishmania* is a protozoan parasite causing leishmaniasis. Parasites are transmitted by the sand fly, and an estimated 350 million people are at risk of infection in over 88 countries worldwide [1]. There are three forms of leishmaniasis: cutaneous, muco-cutaneous, and visceral leishmaniasis. The most common is cutaneous leishmaniasis, caused by over 20 different *Leishmania* spp. and characterized by ulcers or nodules on the skin. Muco-cutaneous leishmaniasis, which is caused by parasites of the *Viannia* subgenus, especially *Leishmania* (*V.*) *braziliensis*, *Leishmania* (*V.*) *panamensis*, and *Leishmania* (*V.*) *guyanensis*, and also *Leishmania amazonensis*, is characterized by a progressive destruction of the mucosa by ulcers that spread from the mouth and nose to the pharynx and larynx. The third form, visceral leishmaniasis which is caused by *Leishmania donovani* and *Leishmania infantum*, is a systemic infection characterized by prolonged fever, anemia, weight loss, hepatosplenomegaly, and, in severe cases, death [2–6]. In Ghana, one of the three ecological zones, arid Northern savanna, epidemiologically lies within the leishmaniasis belt in Africa. There have however been no reports of leishmaniasis in this region of the country. Most reported cases of leishmaniasis in Ghana have come from the Ho district of the Volta Region which is the moist semi-deciduous forest region of Ghana [7]. Cutaneous leishmaniasis (which may include muco-cutaneous, because of problems with diagnosis) is the only kind of leishmaniasis reported in this region. The species of *Leishmania* recorded in Ghana as the causative agent of cutaneous leishmaniasis is *Leishmania major* [8]. More recently, a new member of the *Leishmania enriettii* complex has been detected and isolated in Ghana [9].

The main form of control of leishmaniasis is chemotherapy. The use of the current anti-*Leishmania* drugs are however limited by high toxicity [10]. The treatment of leishmaniasis involves the use of specific anti-*Leishmania* drugs and the aggressive management of any bacteria or parasitic co-infection, hypovolemia (decreased blood volume), and malnutrition [10]. Pentavalent antimonials, the first line treatment for leishmaniasis, have an efficacy dependent on the species of *Leishmania*, the geographical location as well as the clinical presentation of the disease [11]. The antimonials have also been reported to have high toxicity that results in frequent and life-threatening side effects [12]. The second line of treatment, pentamidine and amphotericin B, is limited by high toxicity and/or emergence of resistance [13]. Like the antimonials, the second line of treatment requires parenteral administration. Last but not least, Miltefosine, the only oral treatment for leishmaniasis, is teratogenic and is therefore not suitable for women of childbearing age [14].

Although the promastigote stage of the *Leishmania* parasite is mainly found in the sand fly vector, their ease of handling has made them a very useful tool in the determination of the anti-*Leishmania* activity of compounds and extracts. Also, promastigotes have been reported to be a good option for the screening of compounds whose anti-*Leishmania* activity is not dependent of cell-mediated parasiticidal mechanisms [15]. This is because their activities against the promastigote stage would be representative of their activities against the intracellular amastigotes [15].

In recent times, there have been several reports on the use of medicinal plants for the treatment of various ailments. Medicinal plants like *Zanthoxyum zanthoxyloides* and *Annona senegalensis* have been reported to have some anti-*Leishmania* activity [16, 17]. In our previous study, we isolated three novel tetracyclic iridoid compounds from *Morinda lucida* benth, *Molucidin*, *ML-2-3*, and *ML-F52* and found them to have anti-trypanosome activity [18]. *Leishmania* species belongs to the same kinetoplastid protozoan group with *Trypanosoma* and shares very similar life cycle and organelles such as flagellum and kinetoplast. In this study, we have tested those novel compounds against *Leishmania* spp. and found that two of these three compounds, Molucidin and ML-F52, had activities against *Leishmania* species, suggesting Molucidin and ML-F52 to be promising lead compounds for the treatment of leishmaniasis.

## Materials and methods

### *Leishmania* parasites

The field strain of *L. enriettii* (010) promastigotes, which had been isolated in Ghana, and the lab strain, *Leishmania hertigi* promastigotes, were used in this study because of their availability. Parasites were cultured in vitro according to conditions established previously [19]. Log phase of parasites (1 × 10^6^ parasites/ml) was diluted to a parasitemia of 3 × 10^5^ parasites/ml with M199 medium and used for the drug assay. Estimation of parasitemia was done with the Neubauer’s counting chamber.

### In vitro viacount assay for *L. hertigi* and the Ghanaian field strain (010)

*Leishmania* promastigote forms of *L. hertigi* parasites were seeded and incubated in the presence or absence of (50–0.78 μM) compounds (dissolved in DMSO) for 48 h, after which the Guava reagent for viacount (Millipore) was added to the culture in the ratio of 1:1 and incubated for 30 min. Analysis was performed using the Millipore guava easyCyte 5HT (USA) according to the manufacture’s instruction.

Promastigotes of *Leishmania* 010 were seeded and incubated in the presence or absence of (50–0.78 μM) compounds for 96 h and the minimum inhibitory concentration (MIC) of the compounds determined by microscopy.

### Fluor-microscopy analysis using DAPI

Parasites were incubated for 24 h under appropriate conditions (25 °C) with appropriate concentrations of active compounds, Molucidin and ML-F52. Parasites were then harvested and fixed with 4 % paraformaldehyde at room temperature for 5 min. Washing steps were carried out with PBS and PBST (0.1 % Triton X 100 in PBS) at room temperature for 5 min and 15 min, respectively. The parasites were stained with 4’,6-diamidino-2’-phenylindole dihydrochloride (DAPI) (5 μg/ml DAPI in PBS) for 10 min. After washing steps as above, the slides were mounted using parmafluor mounting reagent and covered with cover slips. The slides were observed under the Olympus fluorescent microscope (Olympus BX53) to detect any phenotypic changes in *L. hertigi* parasites.

## Results and discussion

### Activity of compounds against *Leishmania* parasites

The aim of the study was to investigate the anti-*Leishmania* properties of the three novel tetracyclic iridoid compounds previously found to have anti-*trypanosome* activity. In the determination of the effect of Molucidin, ML-2-3, and ML-F52 on *L. hertigi* and 010, parasites were challenged with 0–50 μM of each compound for 48 and 96 h to determine MIC and the concentration of compounds that inhibited parasite growth by 50 % (IC_50_) by microscopy and fluorescence-activated cell sorting (FACS) analysis, respectively. Molucidin and ML-F52 had anti-*Leishmania* activity against both strains while ML-2-3 had no activity. Molucidin showed anti-*L. hertigi* activity with IC_50_ of 4.24 μM and anti-010 activity with MIC of 4.167 μM (Table 1). Compound ML-F52 showed anti-*L. hertigi* activity with IC_50_ of 3.38 μM and anti-010 activity with MIC of 2.60 μM (Table 1). All three compounds share same side chains, iridoid tetracyclic skeleton, but differ in their functional group region. Molucidin and ML-F52 are esters while ML-2-3 is carboxylic acid. The functional groups may play a key role in the activity of the compounds against *Leishmania*. With respect to toxicity, ML-F52 had a selective index (SI values) in the range of 1.4–5.36 while Molucidin had SI values in the range of 1.67–2.20, respectively (Table 2).

**Table 1:** Anti-*Leishmania* activity of three anti-trypanosome compounds

	Molucidin (μM)	ML-2-3 (μM)	ML-F52 (μM)
IC_50_ after 48 h (*L. hertigi*)	4.24	>50	3.38
MIC after 96 h (010)	4.17	>50	2.60

IC_50_ of amphotericin B (positive control) was 0.1 μg/ml

**Table 2:** Selective index of compounds tested for anti-*Leishmania* activity

		IC50 (μM), 48 h			SI		
		ML-2-2	ML-2-3	ML-F52	ML-2-2	ML-2-3	ML-F52
IC50 of anti-leishmanial activity		4.24	>50	3.38			
Normal skin fibroblasts	NBIRGB	7.11	>50	4.74	1.67	>0.14	1.4
Normal lung fibroblasts	HF-19	14.24	>50	10.94	3.36	>0.28	3.23
Normal lung	Hs-888Lu	9.29	>50	8.89	2.19	>0.19	2.63
Normal liver	Chang Liver	9.34	>50	18.13	2.20	>0.19	5.36

### Effect of compounds on parasite morphology and flagella function

**Fig. 1:**
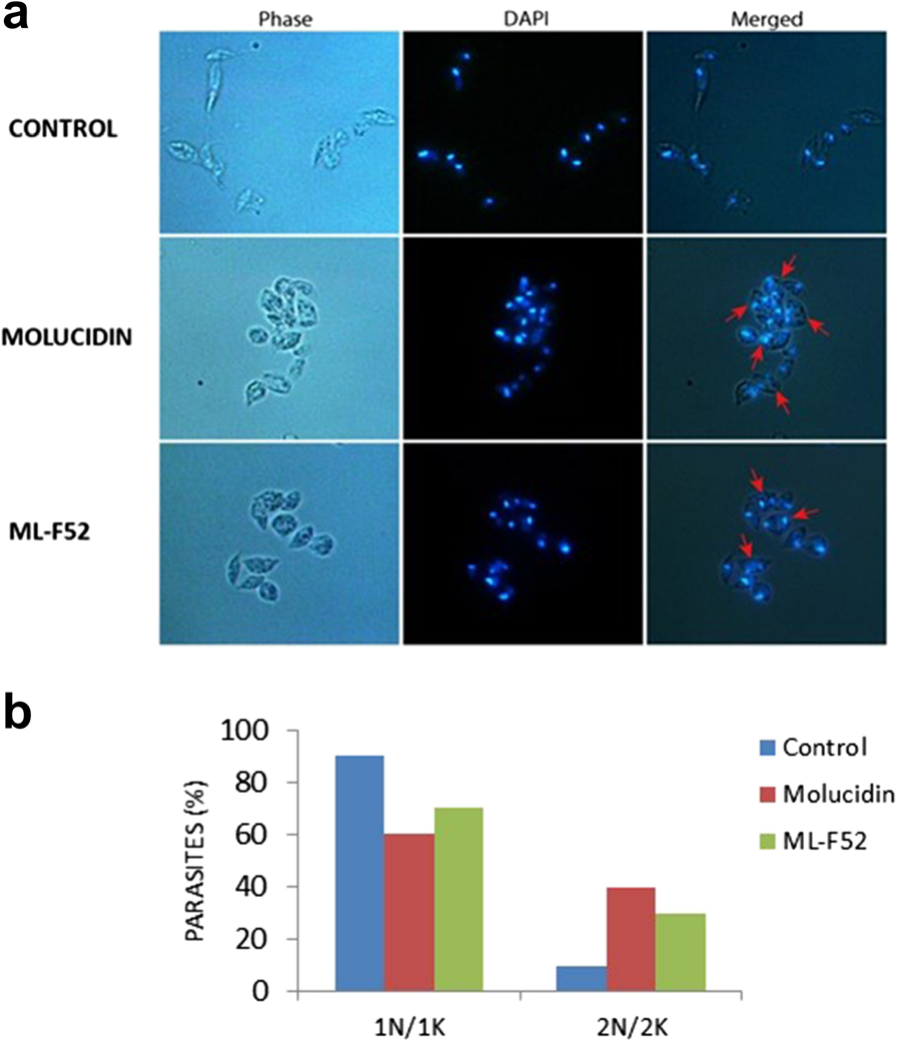
Effect of Molucidin and ML-F52 on the nucleus, kinetoplast, and morphology of the parasite. **a** DAPI staining and fluor-microscope observation of parasites challenged with Molucidin and ML-F52. Both compounds induced short stumpy like parasite without long flagellum in which the cells having two sets of nucleus and kinetoplast in a cell were observed (indicated by *red arrowhead*). **b** Graph showed the ratio of normal cells which has one set of nucleus and kinetoplast (*1N/1K*) and abnormal cells induced by compounds which has two sets of nucleus and kinetoplast in a cell (*2N/2K*). 2N/2K cells were significantly induced by both Molucidin and ML-F52

To investigate the effect of Molucidin and ML-F52 on the morphology and cell division of *L. hertigi*, we performed fluor-microscope observation with DAPI staining after incubation of parasite with 4 μM of Molucidin and ML-F52 for 48 h. Both Molucidin and ML-F52 induced short stumpy form parasite cells (Fig. 1a), in which two sets of nucleus and kinetoplast were observed (2N/2K), while normal cells had a set of nucleus and kinetoplast in each cell (1N/1K). Microscopic counting resulted in different ratios of both populations in Molucidin- and ML-F52-treated *Leishmania* as follows: normal cells (data not shown) and DMSO-treated cells showed 90 % of 1N/1K and 10 % of 2N/2K, while both Molucidin and ML-F52 caused significant increase with 30–40 % of 2N/2K and decrease of 1N/1K cells (Fig. 1b). These results indicated that Molucidin and ML-F52 might inhibit cytokinesis after division of both nuclei and kinetoplast in *Leishmania* parasites since there was also no observable inhibition of nuclear or kinetoplast division nor signs of nuclear and/or kinetoplast disintegration or fragmentation. This result indicated that *Leishmania* parasites challenged with both Molucidin and ML-F52 were able to undergo both nuclei and kinetoplast division but are unable to progress through mitosis to form two distinct daughter cells. The inability of cells to undergo cytokinesis has been shown to cause cell cycle arrest leading to the death of parasites [20, 21]. The inhibition of cytokinesis might imply the inability of cells to replicate in the mammalian host and increase the parasite burden. This may prevent the parasites from over running the host-immune and may give the host the edge to clear the infection [22]. On the other hand, this cell cycle inhibition may lead to the induction of apoptosis-like death in the *Leishmania* cells and may be the mechanism by which both compounds induce death [23, 24]. In fact, we previously found cell cycle alteration and apoptosis induction in Trypanosoma parasite challenged with ML-2-3 and ML-F52. Although there should be further analysis done to elucidate the mechanism of action for these compounds, they might be promising lead compounds for the development of an alternate anti-*Leishmania* drug.

## Conclusion

Molucidin and ML-F52 are promising compounds for the development of novel anti-*Leishmania* drugs. The development of one or both compounds may lead to the development of a safer, cheaper, and relatively more available drug(s) for the treatment of leishmaniasis.

## Abbreviations

FACS, fluorescence-activated cell sorting; DAPI, 4’,6-diamidino-2’-phenylindole dihydrochloride; MIC, minimum inhibitory concentration; IC_50_, compound concentration that inhibits parasite growth by 50 %; SI, selective index; N/K, nucleus and kinetoplast
